# Long non-coding RNAs in the regulation of myeloid cells

**DOI:** 10.1186/s13045-016-0333-7

**Published:** 2016-09-29

**Authors:** Xinyu Tian, Jie Tian, Xinyi Tang, Jie Ma, Shengjun Wang

**Affiliations:** 1 Department of Laboratory Medicine, The Affiliated People’s Hospital, Jiangsu University, Zhenjiang, 212002 China; 2 Institute of Laboratory Medicine, Jiangsu Key Laboratory of Laboratory Medicine, School of Medicine, Jiangsu University, Zhenjiang, 212013 China

**Keywords:** Long non-coding RNAs, Erythrocytes and megakaryocytes, Granulocytes, Monocytes and macrophages, Cell development

## Abstract

Long non-coding RNAs (lncRNAs) have been attracting immense research interests. The relevance of lncRNAs in biological and physiological as well as in pathological processes has increased along with the understanding of their various regulatory mechanisms. Abundant studies have indicated that lncRNAs are involved in the differentiation, proliferation, activation, and initiation of apoptosis in different cell types. However, most studies about the regulating biology of lncRNAs are currently focused on cancer cells. This review is focused on the widely unexplored role of lncRNAs in the cell fate of myeloid cells. In this review, we summarize recent studies that have confirmed lncRNAs to be essential in the development of myeloid cells under normal and pathological conditions.

## Background

### Myeloid cells

Circulating blood cells are all derived from hematopoietic stem cells (HSCs), and there have been at least two alternative patterns describing the hierarchical hematopoiesis. Multi-potential progenitors (MPPs) derived from HSCs give rise to common myeloid progenitors (CMPs) and common lymphocyte progenitors (CLPs). CMPs develop into granulocyte-macrophage progenitors (GMPs), which are the sources of granulocytes, monocytes, and megakaryocyte-erythroid progenitors (MEPs) that can give rise to erythrocytes and megakaryocytes. On the other hand, CLPs differentiate into immune cells, such as T cells, B cells, dendritic cells (DC), and NK cells. Another pattern of hematopoiesis differs by the involvement of intermediate lymphoid-primed multi-potential progenitors (LMPPs) that can differentiate into GMPs and CMPs [1].

During the process of CMPs differentiating into different lineages, protein molecules, such as growth factors, cytokines, and transcription factors (TFs), which label the cell path and participate up to the destiny, comprise the complicated regulatory network [1–12]. Furthermore, non-coding RNA (ncRNA), an additional regulation factor, has joined the regulatory network of myeloid cell development. Recently, regulatory ncRNAs have been further divided into small ncRNAs and emerging long ncRNAs [13–17]. This review is focused on regulatory long non-coding RNAs (lncRNAs) in myeloid cells.

### LncRNAs

#### Expression and regulation of lncRNAs

With the development of gene tiling arrays and transcriptome studies, it has been suggested that only 2 % of the human genome is transcribed and translated into proteins, while nearly 90 % is transcribed into ncRNAs [18]. According to the length of the transcript, ncRNAs are divided into small ncRNAs (<200 nt), such as microRNAs, and long ncRNAs (>200 nt). Initially recognized as “transcriptional noise” in previous RNA sequence studies, lncRNAs are now characterized as functional RNA elements with features of shorter length, containing fewer exons, and expressed at lower levels compared to messenger RNAs (mRNAs) [19–21]. Like for mRNAs, lncRNAs are transcribed by RNA polymerase II. Processing of lncRNAs involves 3′ poly(A) tailing and 5′-end capping as well as splicing. LncRNAs have small open reading frames without any protein-coding potential but can sometimes be associated with ribosomes in the cytoplasm, suggesting an additional role in mRNA metabolism [1]. As of now, over 23,000 human lncRNA genes have been found and are mostly expressed in a cell-, tissue-, or developmental stage-specific manner. Currently, based on the localization of lncRNAs to protein-coding mRNAs, lncRNAs are classified into long intergenic ncRNAs (lincRNAs), enhancer RNAs (eRNAs), antisense lncRNAs, intronic lncRNAs, and transcribed pseudogene lncRNAs [19]. However, in light of the accumulating information on sequencing data and the function of lncRNAs, these arbitrary definitions will need to be redefined.

The expression of lncRNAs can be regulated. As portrayed above, lncRNAs show tissue-, cell-, and developmental stage-specific expression and are differently expressed under physiological or pathological conditions, which indicate that these transcripts can be regulated. This regulation of lncRNAs can occur at the transcriptional level. For example, the expression of intronic lncRNAs derived from intronic sequences is usually linked with host gene expression. However, the expression of lincRNAs is promoter-dependent. Additionally, lncRNAs can also be mediated post-transcriptionally based on their different stability. Stability of lncRNAs is associated with their location, splicing, and final subcellular position. In a recent work by Ayupe et al., global analysis of the stability of lncRNAs mapping to intragenic regions of the human genome showed that antisense lncRNAs are significantly more stable than mRNAs, whereas intronic lncRNAs comprise a more heterogeneous class that consist of both stable and unstable transcripts. In mice, it has also been demonstrated that lincRNAs are more stable compared with intronic lncRNAs in mouse neuronal cells. Additionally, the stability of spliced lncRNAs is much more than non-spliced lncRNAs, and the half-life of lncRNAs in the cytoplasm is longer than lncRNAs localized in the nucleus [22]. Thus, altering the stability of lncRNAs can modulate their functions. MicroRNA let-7i has been recently reported to interact with HOX antisense intergenic RNA (HOTAIR), which is an antisense lncRNA transcribed from the HOX locus, and decrease HOTAIR expression. However, HOTAIR is stabilized when let-7i or Argonaute 2 (AGO2) is suppressed, while it is destabilized by let-7i. Meanwhile, HOTAIR is degraded when human antigen R (HuR) promotes the interaction of let-7i-AGO2 with HOTAIR. In cells with suppressed levels of HuR, HOTAIR is stable and accumulates, which contributes to a scaffold for E3 ubiquitin ligases and their respective ubiquitination substrates [23].

#### Regulatory mechanisms of lncRNAs

**Fig. 1:**
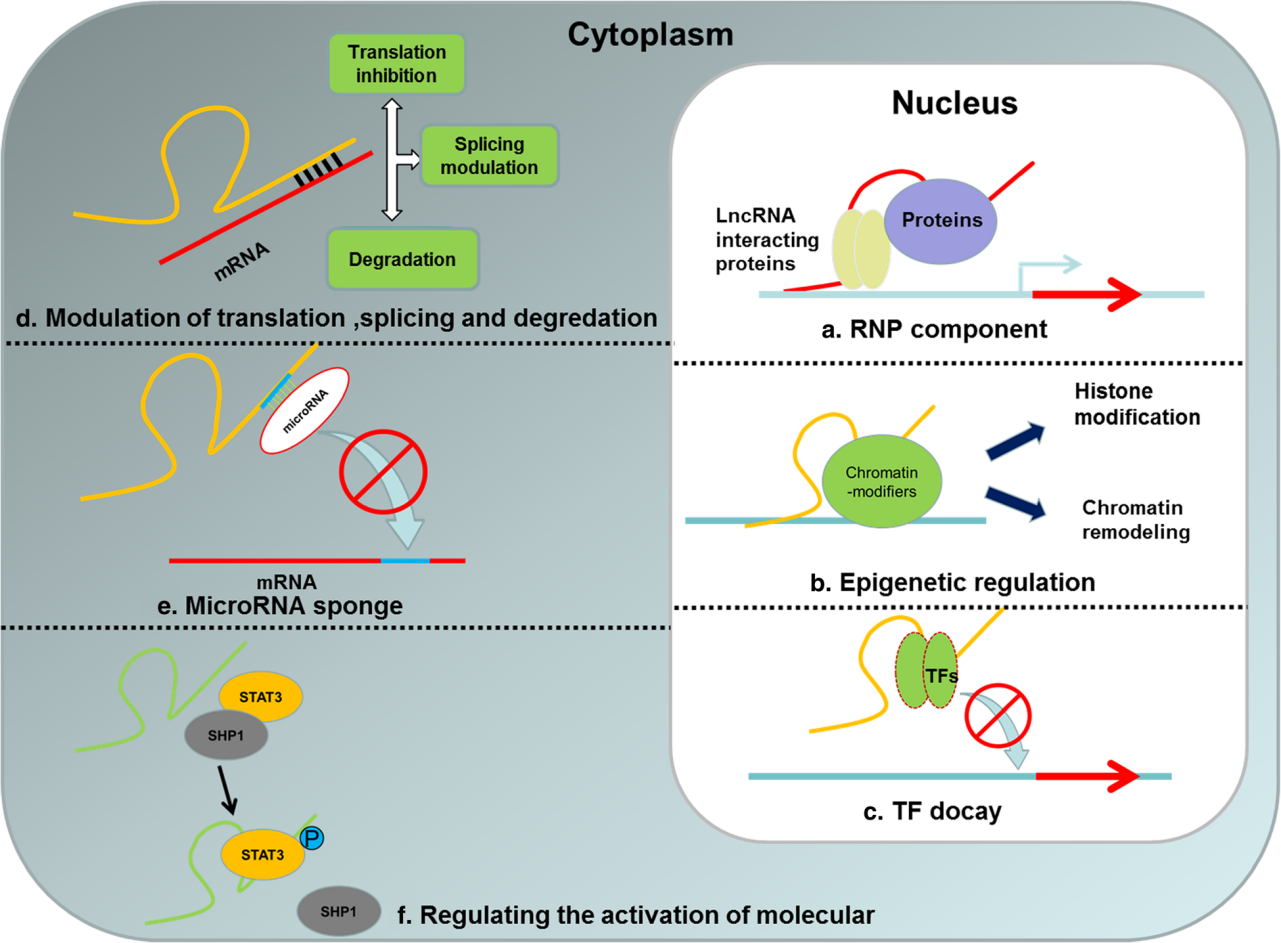
The regulatory mechanisms of lncRNAs located in nucleus and cytoplasm. **a** RNP component. **b** Epigenetic regulation. **c** TF docay. **d** Modulation of translation, splicing, and degradation. **e** MicroRNA sponge. **f** Regulating the activation of molecular

The regulatory mechanisms of lncRNAs are diverse. LncRNAs are capable of interacting with RNA as well as DNA and proteins which attach lncRNAs with various molecular functions transcriptionally and post-transcriptionally. Nuclear lncRNAs can recruit chromatin-modification factors to suppress or activate different loci so as to regulate gene expression epigenetically [24–28]. LncRNAs can also assemble transcriptional activators or repressors to mediate gene transcription. In addition to regulating gene expression, several lncRNAs in the nucleus have been demonstrated to be essential for organizing distinct nuclear structures [29–33]. At the post-transcriptional level, lncRNAs regulate gene expression by mediating translation and enhancing the stability of partially complementary mRNAs. Additionally, lncRNAs can also interfere with RNA-binding proteins to influence splicing and translation as well as regulate the activity and location of the proteins. Finally, lncRNAs can act as a sponge for endogenous microRNAs (miRNAs) [1, 19]. The detailed regulatory mechanisms of lncRNAs are listed in Fig. 1.

Accumulating evidence has indicated that lncRNAs are crucial regulators of the development, differentiation, and proliferation of multiple cells. However, the majority of studies about lncRNAs regulating cell biology are focused on their effect on various cancer cells, while their importance during the development of myeloid cells is also worthy of attention and is the topic of this review.

### LncRNAs in erythrocytes and megakaryocytes

The expression of lncRNAs is different under physiological and pathological conditions, indicating that they may play crucial biological functions. Over 400 putative lncRNAs have been identified during mouse erythropoiesis by an RNA-seq analysis. Thus, lncRNAs play an important role during erythropoiesis. A recent study, by using RNA-seq, has identified 1109 polyA+ lncRNAs in murine megakaryocytes, erythroblasts, and megakaryocyte-erythroid precursors, in which most erythro-megakaryocytic lncRNAs (~75 %) are transcribed from promoters and the other 25 % from enhancers. The following analysis indicated that a majority of these lncRNAs are regulated by crucial TFs, such as GATA1 and TAL1. While these erythroid lncRNAs show dramatic conservation in different mouse strains, only 15 % of mouse lncRNAs are expressed in the human. An RNAi assay was performed on 21 abundant lncRNAs in mouse erythroid precursors and 7 lncRNAs, including Erytha, Ggnbp2os, Bloodlinc, Galont, Redrum, Lincred1, and Scarletltr, knockdown blocked erythroid maturation [34].

### LincRNA-EPS

LincRNA-erythroid pro-survival (EPS) is a PolII transcript located in the nucleus with the length of 2531 nt and contains four exons and three introns in mouse Ter119+ cells. LincRNA-EPS is highly and specifically expressed in terminally differentiating erythroblasts. It has been demonstrated that lincRNA-EPS has a potent anti-apoptotic activity because knockdown of lincRNA-EPS resulted in apoptosis during the terminal differentiation of erythroid cells and blocked the proliferation of erythroid progenitors. Meanwhile, overexpression of lincRNA-EPS can prevent erythroid progenitor cells from undergoing apoptosis caused by Epo deprivation. To confirm which part of linRNA-EPS mediates the apoptosis of erythroid cells, a structure-functional analysis was performed in which different regions of lincRNA-EPS were truncated from the 5′- to 3′-end and then transduced into Lin− fetal liver cells. Interestingly, lincRNA-EPS with 5′ truncation can still suppress apoptosis whereas lincRNA-EPS with 3′ truncation loses this anti-apoptosis activity. Thus, lincRNA-EPS possesses its anti-apoptotic activity via the 500-nt sequence at the 3′-end. This modulation of apoptosis of erythroid progenitors by lincRNA-EPS is partly through the inhibition of Pycard expression, which is capable of encoding the protein to activate caspases during apoptosis [35–37]. Nevertheless, the specific mechanism is still left to be uncovered.

### Shlnc-EC6

Erythroid enucleation is important for the differentiation of mammalian erythrocytes, as excluding the nucleus from red cells allows for a higher concentration of hemoglobin in the blood. Shlnc-EC6 is a lncRNA involved in erythroid differentiation. The work of Wang et al. has demonstrated that shlnc-EC6 knockdown in erythroid progenitor and hematopoietic stem cells (FLEPHSCs) purified from mouse fetal liver is capable of apparently inhibiting erythroid enucleation. It is also confirmed that shlnc-EC6 suppresses Rac1 expression by directly binding the 3′UTR of Rac1 mRNA. Thus, shlnc-EC6 knockdown results in increased expression of Rac1 and the up-regulated activation of downstream PIP5K which leads to the inhibition of enucleation in cultured mouse fetal erythroblasts. This finding indicates that shlnc-EC6 acts as a post-transcriptional regulator to modulate mouse erythropoiesis through the Rac1/PIP5K signaling pathway [38].

### AlncRNA-EC7

It has been shown that the alncRNA-EC7 locus in fetal erythroblasts is in a 6.9-kb region of widespread H3K4me1 and H3K27Ac markings centered around a 5.2-kb region of open chromatin bound by RNA Pol II, GATA1, TAL1, and KLF1, hallmarks of a large enhancer. At this site, bidirectionally transcribed long RNAs are spliced and polyadenylated to RNAs crossing shorter regions concentrating in the poly(A)− fraction. AlncRNA-EC7 is located in the nucleus and its enhancer site is conserved in humans. Additionally, alncRNA-EC7 is an enhancer of SLC4A1, the coding gene of BAND3, which is the major anion exchanger of the erythrocyte membrane and a mutation of which can lead to hemolytic anemias. Knockdown of alncRNA-EC7 from the enhancer leads to significantly decreased expression of neighboring BAND3. Thus, consistent to the co-expression between alncRNA-EC7 and BAND3, alncRNA-EC7 is capable of enhancing the expression of BAND3 in cis. Mapping of long-range chromatin interactions associated with CCCTC-binding factor (CTCF) and RNA Pol II in K562 cells reveals chromatin looping between the enhancer site and the BAND3 promoter-proximal region. Taken together, alncRNA-EC7 mediates erythropoiesis partly by regulating enhancer looping to activate the BAND3 locus [39, 40].

### MONC and MIR100HG

LincRNAs MONC and MIR100HG, which are mainly localized in the nucleus, are highly expressed in acute megakaryoblastic leukemia (AMKL) blasts. The expression of these two transcripts is correlated with corresponding miR-99a/100~125b clusters. An ShRNA-induced loss-of-function study suggests MONC or MIR100HG knockdown inhibits leukemic growth of AMKL cell lines and cells from primary patient samples. In the following investigation, a lentiviral lncRNA vector was used to ectopically express lincRNAs without perturbing their secondary structure. The results suggested that ectopic MONC expression in cord blood (CB) CD34+-HSPCs leads to the expansion of immature erythroid precursors and a decrease of granulocytic precursors. These results reveal that MONC interferes with hematopoietic lineage decisions and enhances the proliferation of immature erythroid progenitor cells [41].

### LncRNAs in granulocytes

Granulocytes are essential for host defense against invading pathogens. Augmentation of neutrophil function is beneficial for many pathologic conditions. Here, we summarize the regulation of lncRNAs in the development and function of granulocytes and their regulatory mechanisms.

### HOTAIR

In the human genome, there are four HOX clusters, HOXA, HOXB, HOXC, and HOXD. These four HOX clusters are probably derived from a single ancestral cluster and are conserved regulators of embryonic patterning and development [42, 43]. HOXA and HOXB have appeared as the key transcriptional regulators in hematopoiesis [44, 45]. For example, HOXA9 and HOXA10 expressed by mature neutrophils can mediate the transcription of genes associated with the phagocytic function of neutrophils [46–48]. Additionally, HOX genes also promote the pathogenesis of acute leukemia and the self-renewal ability of leukemia stem cells [49]. It is indicated that, in the intergenic region of human HOX genes, lincRNAs, which are demonstrated to be direct regulators of cellular functions, show much more transcription activity than microRNAs, such as miRNA-10 and miRNA-196 [50, 51]. Among these lincRNAs, HOTAIR is the first to be defined.

HOTAIR (Hox transcript antisense intergenic RNA) is a lincRNA with a length of 2158 nt. Although it is located in the HOXC gene cluster, HOTAIR can modulate the remote HOXD gene cluster and a network of discrete non-HOX gene loci by recruiting elements of the histone-modifying PRC2 and LSD1 complex [52–54]. However, a majority of previous studies about HOTAIR, which has been indicated as a negative prognostic marker, are focused on its role in the cell biology of various cancer cells and little is known about the regulation of HOTAIR in myeloid cells. Recently, the expression of HOTAIR has been demonstrated to be significantly up-regulated in AML-de novo patients compared to AML-CR patients and normal controls, and the higher level of HOTAIR in AML patients is positively correlated with the NCCN high-risk group. In addition, HOTAIR knockdown is capable of inhibiting the proliferation of AML cells [55].

### HOTAIRM1

In addition to HOTAIR, HOTAIRM1 (HOX antisense intergenic RNA myeloid 1) is indicated to be one of the three other lincRNAs that exist in the intergenetic region of HOX gene clusters. HOTAIRM1, transcribed by RNA polymerase II, is located between the human HOXA1 and HOXA2 genes, and its expression is increased during granulocytic differentiation. HOTAIRM1, which shows myeloid-specific expression, is transcribed antisense to HOXA genes and originates from the same CpG island that embeds the start of HOXA1. In the process of induced granulocytic differentiation, HOTAIRM1 has been shown to be up-regulated and the most prominently expressed intergenic transcript. During retinoic acid (RA)-induced granulocytic differentiation of NB4 cells, the up-regulation of HOTAIRM1 is based on the expression of myeloid cell development factors targeted by RA signaling. Knockdown of HOTAIRM1 significantly inhibits the expression of HOXA1 and HOXA4 and blunts induction of transcripts for the myeloid differentiation genes, such as CD11b and CD18, in the process of RA-induced granulocytic differentiation. However, HOTAIRM1 knockdown does not impact the more remote HOXA genes. These findings suggest that HOTAIRM1 plays a role in myelopoiesis through modulation of gene expression in the HOXA cluster [50]. The following work of Zhang et al. demonstrated a potential mechanism in which HOTAIRM1 influences the all trans retinoid acid (ATRA)-induced granulocytic differentiation. They find that knockdown of HOTAIRM1 apparently delays morphological granulocytic maturation during ATRA-induced granulocytic differentiation of NB4 cells and significantly increases the proliferation of immature cells at the G1/S phase transition during ATRA-induced cell cycle arrest. Along with resistance to arrest of cell cycle progression, HOTAIRM1 knockdown alters ATRA-induced changes in CD11c and CD49d expression. The coupling of cell cycle progression with temporal dynamics in the expression patterns of these integrin genes suggests a regulated switch to control the transit from the proliferative phase to granulocytic maturation. Furthermore, ITGAX is among a small number of genes showing perturbation in transcript levels upon HOTAIRM1 knockdown even without ATRA treatment, suggesting a direct regulatory pathway [56]. These results indicate that HOTAIRM1 provides a regulatory link in myeloid maturation by modulating integrin-controlled cell cycle progression at the gene expression level. Additionally, in our own research about lncRNA, it is suggested that HOTAIRM1 is associated with the immunosuppression of granulocytic myeloid-derived suppressor cells (G-MDSCs) in lung cancer patients. All of these facts indicate the importance of HOTAIRM1 in regulating the cell biology of granulocytes.

Additionally, in ATRA-induced differentiation of acute promyelocytic leukemia (APL) cells, it has been revealed that PU.1 constitutively binds to the regulatory region of HOTAIRM1, which leads to the transactivation of the regulatory region of HOTAIRM1. Further analysis suggests that two PU.1 motifs located around +1100 bp downstream of the transcriptional start site of the HOTAIRM1 promoter are responsible for the PU.1-induced transactivation. Furthermore, ATRA-induced HOTAIRM1 is PU.1-dependent, and overexpression of PU.1 significantly increased HOTAIRM1 levels. Additionally, HOTAIRM1 expression is decreased in APL cells, which is attributed to the reduced PU.1 expression rather than the repression by PML-RARα via direct binding [57].

### NEAT1

Nuclear paraspeckle assembly transcript 1 (NEAT1), which is a nuclear-restricted long ncRNA, has two isoforms: 3.7-kb NEAT1-1 and 23-kb NEAT1-2. NEAT1 has recently been identified as a crucial component of a subnuclear structure named the paraspeckle, which has been shown to regulate gene expression through retaining mRNA for editing in the nucleus. It has also been suggested that NEAT1 controls several biological processes including cellular differentiation [58, 59]. In APL, an aberrant chromosomal translocation that fuses a portion of the promyelocytic leukemia (PML) gene with the retinoic acid receptor α (RARα) gene and subsequent expression of the PML-RARα oncoprotein causes a differentiation blockade at the promyelocytic differentiation stage. It is confirmed that the expression of NEAT1, which consists of NEAT1-1 and NEAT1-2, is dramatically decreased in PBMCs of APL patients compared to normal granulocytes. This down-regulation of NEAT1 has been demonstrated to be caused by PML-RARα and can be restored by ATRA. However, when NEAT1 expression is blocked, ATRA cannot continue to promote the differentiation of granulocytes. Furthermore, NEAT1 can also mediate the stress response [60].

### HOXA-AS2

HOXA cluster antisense RNA 2 (HOXA-AS2) is a long intergenic ncRNA between and antisense to the HOXA3 and HOXA4 genes in the HOXA cluster. This transcript is expressed in NB4 PML cells and human peripheral blood neutrophils. The current results indicate that HOXA-AS2 plays a role in regulating cell survival through suppressing apoptosis. In NB4 cells stimulated with all trans retinoic acid (ATRA), the expression of HOXA-AS2 is dramatically up-regulated, and in primary peripheral blood neutrophils, HOXA-AS2 expression is induced by interferon gamma (IFN-γ) and TNF-α. During the proliferation of NB4 cells, the number of viable cells is decreased post HOXA-AS2 knockdown by shRNA, while the proportion of apoptotic cells is increased. The increase in death of HOXA-AS2 knockdown cells is accompanied by elevated TNF-related apoptosis-inducing ligand (TRAIL) levels, but ATRA-induced NB4 cells treated with TRAIL do not show an increase in HOXA-AS2 expression. These results demonstrate that ATRA induction of HOXA-AS2 inhibits ATRA-induced apoptosis, possibly through a TRAIL-mediated pathway. HOXA-AS2-mediated negative regulation thus contributes to the fine-tuning of apoptosis during ATRA-induced myeloid differentiation in NB4 cells [61].

### PVT1

The lncRNA PVT1 is located on chromosome 8q24, a location shared with the well-known oncogene c-myc. Chromosome 8q24 has an equivalent in mice (chromosome 15), which is the most commonly recurring abnormality in PML-RARα transgenic mice, and it cooperates with PML-RARα to accelerate the development of myeloid leukemia. By comparing PVT1 expression in granulocytes from healthy donors and APL patients, it has been shown that PVT1 is significantly up-regulated in APL patient samples. In the process of ATRA-induced granulocytic differentiation, treatment of APL cells with ATRA represses both PVT1 expression and the expression of c-myc mRNA, while knockdown of MYC in NB4 cells leads to PVT1 down-regulation. These data suggest that PVT1 may be regulated by MYC and is involved in the proliferation of APL cells. Further investigations have revealed that PVT1 knockdown has no effect on c-myc RNA but leads to the suppression of the MYC protein level in NB4 cells. At the same time, NB4 cells with PVT1 knockdown have a lower survival rate than those in the control group, which suggests that knockdown of PVT1 impairs the proliferation of APL cells [62].

### EGO

Eosinophil granule ontogeny (EGO) is an intronic lncRNA derived from the inositol trisphosphate receptor type 1 (ITPR1) gene locus. EGO is a conserved primary transcript among humans, mice, and chickens. This lncRNA is up-regulated with the maturation of eosinophils. Biochemical experiments have indicated that EGO is non-coding because it is not associated with ribosomes and has no conserved open reading frames (ORFs). Interestingly, a loss-of-function study suggested that knockdown of EGO compromises the expression of several proteins that are important for eosinophil development, such as major basic protein and eosinophil-derived neurotoxin, revealing the functional importance of EGO in eosinophilopoiesis [63].

### Morrbid

Precise regulation to the lifespan of neutrophils, eosinophils, and “classical” monocytes is important for enhancing immune responses against chronic inflammation. It has recently been confirmed that a lncRNA, termed myeloid RNA regulator of Bim-induced death (Morrbid), mediates the survival of neutrophils, eosinophils, and classical monocytes in response to pro-survival cytokines. Morrbid is a conserved transcript across species, contains five exons, and is polyadenylated. It is localized predominately in the nucleus bound to chromatin. Morrbid is highly and specifically expressed by mature eosinophils, neutrophils, and classical monocytes in both mice and humans. Morrbid regulates the lifespan of eosinophils, neutrophils, and monocytes through promoting the enrichment of the PRC2 complex at the promoter of Bcl2L11 (Bim), a neighboring pro-apoptotic gene of Morrbid, and in turn, accelerating the deposition of H3K27-me3 to maintain this gene in a poised state. This Morrbid-regulated process occurs in cis, enabling allele-specific control of Bcl2L11 transcription. Thus, in these highly inflammatory cells, changes in Morrbid levels provide a locus-specific regulatory mechanism that allows for rapid control of apoptosis in response to extracellular pro-survival signals. Additionally, Morrbid may be a potential therapeutic target for the treatment of inflammatory disorders characterized by aberrant short-lived myeloid cell lifespan because it is dysregulated in patients with hypereosinophilic syndrome [64].

### LncRNAs in monocytes and macrophages

Monocyte-macrophages are crucial for the modulation of physiological and pathological processes such as inflammation, tissue damage and repair, metabolism, and pathogen response. Monocytes derived from bone marrow migrate to tissues and organs along with blood circulation and then develop into macrophages. Macrophages are able to differentiate and polarize into the classically activated M1 phenotype or alternatively activated M2 phenotype. Generally, M1 macrophages play important roles in host defense and inflammation, while M2 macrophages play a part in tissue repair [65]. M1 and M2 show distinct gene profiles that are regulated by specific signaling cascades, TFs, and epigenetic factors [66]. Currently, a variety of lncRNAs have been demonstrated to impair the function and development of monocyte-macrophages.

### PACER

In the PMA-induced human monocyte-macrophage differentiation system, it is demonstrated that PACER, an antisense long ncRNA located in the nucleus, is expressed in the upstream region of COX-2. CTCF/cohesin promotes PACER expression by establishing a chromatin domain characterized by increased H3K4 methylation and H4K8 acetylation and decreased H4K20 trimethylation, thus forming a permissive chromatin environment and protecting the COX-2 regulatory region from surrounding repressive chromatin. COX-2 expression is accelerated by PACER in PMA-driven human monocyte-macrophage differentiation with subsequent LPS stimulation through diverse mechanisms. It has been shown that lncRNA PACER is capable of sequestering the repressive NF-kB p50 homodimer from binding to the promoter of COX-2 and facilitating the formation of the active p50–p65 form of NF-kB in the promoter region of COX-2. At the same time, PACER enhances recruitment of the p300 histone acetyltransferase (HAT) and RNAP II pre-initiation complex to increase histone acetylation and induce COX-2 transcription [67].

### Lnc-MC

A long non-coding monocytic RNA (lnc-MC) exhibits increased expression during the differentiation from monocyte to macrophage of THP-1 and HL-60 cells as well as CD34+ hematopoietic stem/progenitor cells (HSPCs). Lnc-MC is transcriptionally activated by PU.1 which can weaken the repressive effect of miR-199a-5p on lnc-MC expression and function as well as promote the monocyte/macrophage differentiation. Lnc-MC facilitates the monocyte/macrophage differentiation of THP-1 cells and CD34+ HSPCs through sequestering miR-199a-5p and alleviating repression on the expression of activin A receptor type 1B (ACVR1B) which is a key regulator of monocyte/macrophage differentiation. Thus, the two PU.1-regulated ncRNAs, lnc-MC and miR-199a-5p, have opposing roles in monocyte/macrophage differentiation [68].

### LincRNA-Cox2

In mouse macrophages, lincRNA-Cox2 is located 50-kb downstream from the Cox2 (Ptgs2) gene appearing in both the cytosolic and nuclear compartments and its expression is induced by TLR ligands in a MyD88- and NF-kB-dependent manner. LincRNA-Cox2 has been shown to inhibit expression of 787 genes in non-stimulated bone marrow-derived macrophages (BMDMs) and increase the expression of 713 genes in BMDM following stimulation with the synthetic bacterial triacylated lipopeptide Pam3-Cys-Ser-Lys4 (Pam3CSK4). Genes involved in the immune response, such as CCL5 and interleukin (IL)-6, were shown to be included. LincRNA-Cox2 suppresses gene expression through interacting with hnRNP-A/B and hnRNP-A2/B1, which are members of a family of multifunctional RNA-binding proteins that are known to have a role in the processing of precursor mRNA as well as in regulating gene expression [69]. A recent study has indicated that in intestinal epithelial cells, lincRNA-Cox2 mediates the transcription of IL-12b induced by TNF-a through modulating Mi-2/NuRD-mediated epigenetic histone modification [70]. As the most highly TLR ligand-induced lincRNA in macrophages, lincRNA-Cox2 has recently been found to be an early-primary inflammatory gene regulated by NF-kB signaling in murine macrophages. After macrophages are stimulated by LPS, lincRNA-Cox2 is assembled into the switch/sucrose non-fermentable (SWI/SNF) complex which is capable of mediating the assembly of NF-kB subunits to the SWI/SNF complex and ultimately, SWI/SNF-associated chromatin remodeling and transactivation of the late-primary inflammatory-response genes in macrophages in response to microbial challenge. Therefore, this indicates a new regulatory role for NF-kB-induced lincRNA-Cox2 acting as a coactivator of NF-kB for the transcription of late-primary response genes in innate immune cells through modulation of epigenetic chromatin remodeling [71].

### THRIL

It has been shown that in human THP1 macrophages, THRIL, which has been identified as an antisense lncRNA (overlapping BRI3BP), can up-regulate TNF transcription through forming a complex with hnRNPL and binding to the promoter of TNF. Pam3Csk4 decreases THRIL expression in THP1 macrophages indirectly by inducing the release of TNFα. THRIL knockdown induces the decreased expression of 444 genes in THP1 macrophages. In addition, THRIL knockdown blocks the different expression of 317 genes out of 618 genes observed in THP1 macrophages stimulated with Pam3CSK4, including multiple inflammatory genes such as IL6, CXCL8, CXCL10, CCL1, and CSF1 [72].

### Lnc-DC

By analysis of the expression profile of lncRNAs during monocytes differentiating into dendritic cells, the level of lnc-DC has been identified to be especially increased. Additionally, PU.1 which is a key regulator of DC differentiation induces the exclusive expression of lnc-DC in human cDCs through binding to the promoter region. By using chromatin ChIP-seq and multisite ChIP-qPCR, high occupancy of Pol II along with increased histone H3-lysine-4 trimethylation (H3K4me3) and histone H3-lysine-27 acetylation (H3K27ac) around the lnc-DC transcription start site (TSS) in Mo-DCs have been identified. Lnc-DC knockdown during Mo-DC differentiation decreases the expression of CD40, CD80, CD86, and HLA-DR on the surface of cells and affects their capacity to uptake antigen and activate CD4+ T cells, while overexpression of lnc-DC has the opposite effect. Lnc-DC regulates Mo-DC differentiation by directly interacting with STAT3, which is a crucial transcription factor of Mo-DC differentiation in cytoplasm, to prevent Y705 dephosphorylation of STAT3 by SHP1 [73].

However, in a recent work of Dijkstra and Ballingall, it is mentioned that the so-called mouse lnc-DC ortholog gene in the study by Wang et al. has already been designated “Wdnm1-like” and is able to encode a small secreted protein. Wdnm1-like is found in mammals; however, the incapacitation of the Wdnm1-like open reading frame is definitely rare with all investigated primates except for hominids having an intact ORF. Thus, it is supposed that the human lnc-DC transcript might only represent a non-functional relatively young evolutionary remnant of a protein-coding locus. Nevertheless, if the conclusions by Wang et al. on their human model are correct, then current knowledge regarding the Wdnm1-like locus suggests an intriguing combination of different functions mediated by transcript and protein in the maturation of several cell types at some point in evolution [74].

### LncRNA-E330013P06

As is known, macrophages mediate the accelerated inflammation associated with diabetes complications. Nevertheless, the specific molecular mechanisms are still unknown. In a study by Reddy et al, RNA sequencing was used to detect the transcripts of bone marrow macrophages isolated from diabetic db/db mice and control db/+ mice. The RNA sequencing results indicated that diabetes promotes the polarization of macrophages into M2, which is a pro-inflammatory, pro-fibrotic, and dysfunctional alternatively activated macrophage phenotype, through TFs associated with macrophage function. Bioinformatics analysis of RNA-seq data identify that diabetes can change the expression of several long ncRNAs. Among these lncRNAs, lncRNA E330013P06 is significantly up-regulated in M2 macrophages. Furthermore, human lncRNA E330013P06 genomic organization is similar to the mouse gene and is a host gene for miR-143 and miR-145 which are implicated in cancer, vascular disease, and insulin resistance. It is supposed that sustained overexpression of lncRNA E330013P06 induces the expression of pro-inflammatory and pro-atherogenic genes, such as IL-6, CCL2, CD36, and PTGS2, in macrophages, enhances responses to inflammatory signals, and promotes foam cell formation. However, silencing lncRNA E330013P06 inhibits the expression of inflammatory genes induced by diabetic stimulation. These results confirm the crucial functional roles for lncRNA E330013P06 in macrophages [66].

### TCONS_00019715

In a recent work, by using a microarray, researchers have detected the expression profile of lncRNAs in human monocyte-derived macrophages (MDMs) polarized towards M(IFN-γ + LPS) or M(IL-4) phenotypes. When compared to primary MDMs, expression of 9343 lncRNAs and 4592 lncRNAs are deregulated in the M(IFN-γ + LPS) and M(IL-4) groups, respectively. The following RT-qPCR data are similar to the microarray results. Furthermore, the expression of lncRNA TCONS_00019715 in M(IFN-γ + LPS) macrophages is found to be much higher than in M(IL-4) macrophages. Additionally, the level of TCONS_00019715 is apparently increased when M(IL-4) is converted to M(IFN-γ + LPS), while it is decreased when M(IFN-γ + LPS) is converted to M(IL-4). TCONS_00019715 knockdown down-regulates the expression of M(IFN-γ + LPS) markers and promotes the expression of M(IL-4) markers in THP-1 cells induced by IFN-γ and LPS. These data show lncRNAs play important roles in regulating macrophage polarization [75].

## Conclusions

In the cell fate of myeloid cells, abundant transcriptional factors (TFs) have been demonstrated to play significant roles, creating a complicated regulation network constructed from progenitors to differentiated blood cells. In recent years, with the redefinition and investigation of ncRNAs, long ncRNAs have emerged as essential modulators of cellular biology via various mechanisms. However, the investigation of lncRNAs is associated with great challenges due to the large profiling list and low evolutionary sequence conservation of lncRNAs. In this review, we focus on the regulation of lncRNAs in the development of myeloid cells (Tables 1 and 2). As summarized above, lncRNAs are closely involved in the development of erythrocytes, megakaryocytes, granulocytes, monocytes, and macrophages via diverse regulatory mechanisms (Fig. 1). Among these mechanisms, lncRNAs, acting as endogenous miRNA sponges or competing endogenous RNAs (ceRNAs), are attracting more and more attention which is helpful in characterizing the function of these RNA transcripts. All of these findings make lncRNAs great candidates for participating in finely tuned regulation in the development of myeloid cells.

**Table 1:** LncRNAs involved in the development of myeloid cells

LncRNAs	Model system	Observation	Refs
LncRNAs in erythrocytes and megakaryocytes			
LincRNA-EPS	Mouse Ter119+ cells	LincRNA-EPS regulates apoptosis during the terminal differentiation of erythroid cells and blocks the proliferation of erythroid progenitors partly through inhibiting Pycard expression	[35, 37]
Shlnc-EC6	Erythroid progenitor and hematopoietic stem cells (FLEPHSCs) purified from mouse fetal liver	Shlnc-EC6 knockdown results in increased expression of Rac1 and the up-regulated activation of downstream PIP5K which leads to the inhibition of enucleation in cultured mouse fetal erythroblasts	[38]
AlncRNA-EC7	Differentiating mouse fetal liver red blood cells/K562 cells	AlncRNA-EC7 mediates erythropoiesis partly by regulating enhancer looping to activate the BAND3 locus	[39, 40]
MONC MIR100HG	Cord-blood (CB) CD34+-HSPCs	MONC/MIR100HG knockdown inhibits leukemic growth of AMKL cell lines and primary patient samples; MONC interferes with hematopoietic lineage decisions and enhances the proliferation of immature erythroid progenitor cells	[41]
LncRNAs in granulocytes			
HOTAIR	Human AML cells Lung cancer cells	HOTAIR is up-regulated in acute myeloid leukemia and that indicates a poor prognosis; HOTAIR knockdown is capable of inhibiting the proliferation of AML cells	[54, 55]
HOTAIRM1	ATRA-induced NB4 cells	HOTAIRM1 plays a role in the myelopoiesis through modulation of gene expression in the HOXA cluster and integrin-controlled cell cycle progression	[50, 56]
NEAT1	Acute promyelocytic leukemia cells	ATRA could not continue to promote the differentiation of granulocytes after NEAT1 blockade	[60]
HOXA-AS2	ATRA-induced NB4 cells	HOXA-AS2-mediated negative regulation thus contributes to the fine-tuning of apoptosis during myeloid differentiation	[61]
PVT1	ATRA-induced NB4 cells	Knockdown of PVT1 leads to the suppression of the MYC protein level and impairs the proliferation of APL cells	[62]
EGO	IL-5 treated CD34+ hematopoietic progenitors	EGO is highly expressed in mature of eosinophils; knockdown of EGO compromises the expression of several proteins that are important for eosinophil development	[63]
Morrbid	Human/mouse neutrophils, eosinophils and monocytes	Morrbid integrates extracellular signals to control the lifespan of eosinophils, neutrophils, and monocytes through allele-specific suppression of Bcl2l11	[64]
LncRNAs in monocytes and macrophages			
PACER	PMA- and LPS-stimulated human U937 monocytic cell line	PACER is expressed upstream of the Cox2 promoter and positively regulates COX2 production; PACER binds to and drives the release of the repressive p50 dimer of NF-kB from the Cox2 promoter	[67]
Lnc-MC	Differentiation from monocyte tomacrophage of THP-1, HL-60, HSPCs	Lnc-MC facilitates the monocyte/macrophage differentiation through sequestering miR-199a-5p and alleviating repression on the expression of ACVR1B	[68]
LincRNA-Cox2	Pam3CSK 4-stimulated mouse bone marrow-derived macrophages	LincRNA-Cox2 suppresses gene expression through interacting with hnRNP-A/B and hnRNP-A2/B1, modulating histone modification and epigenetic chromatin remodeling	[70, 71]
THRIL	Pam3CSK 4 -stimulated human THP1 macrophages	THRIL knockdown blocks the different expression of multiple inflammatory genes in THP1 macrophages stimulated with Pam3CSK4	[72]
Lnc-DC	Differentiation of human and mouse dendritic cells	Lnc-DC promotes phosphorylation and activation of STAT3 by blocking its dephosphorylation by SHP1	[73]
LncRNA-E330013P06	Mouse/human bone marrow macrophages	LncRNA E330013P06 is significantly up-regulated in M2 macrophages and its overexpression induces the expression of pro-inflammatory and pro-atherogenic genes in macrophages	[74]
TCONS_00019715	Human monocyte-derived macrophages (MDMs) polarized towards M(IFN-γ + LPS) or M(IL-4) phenotypes	TCONS_00019715 knockdown down-regulates the expression of M(IFN-γ + LPS) markers and promotes the expression of M(IL-4) markers in THP-1 cells induced by IFN-γ and LPS	[75]

**Table 2:** The expressing stage and associated clinical studies of lncRNAs

LncRNAs	Expressing stage	Clinical study	Refs
LncRNAs in erythrocytes and megakaryocytes			
LincRNA-EPS	Terminal erythrocyte	–	[35, 37]
Shlnc-EC6	Erythroid progenitors	–	[38]
AlncRNA-EC7	Erythroid progenitors	–	[39, 40]
MONC/MIR100HG	Erythroid precursors	Acute megakaryoblastic leukemia	[41]
LncRNAs in granulocytes			
HOTAIR	Progranulocytes	Acute myelocytic leukemia Lung cancer	[54, 55]
HOTAIRM1	Neutrophils	–	[50, 56]
NEAT1	Progranulocytes	Acute promyelocytic leukemia	[60]
HOXA-AS2	Progranulocytes/Neutrophils	–	[61]
PVT1	Progranulocytes	Acute promyelocytic leukemia	[62]
EGO	Eosinophils	–	[63]
Morrbid	Neutrophils/eosinophils/monocytes	Hypereosinophilic syndrome	[64]
LncRNAs in monocytes and macrophages			
PACER	Monocytes	–	[67]
Lnc-MC	Monocytes/macrophages	–	[68]
LincRNA-Cox2	Macrophages	–	[70, 71]
THRIL	Macrophages	–	[72]
Lnc-DC	DCs	–	[73]
LncRNA-E330013P06	M2 macrophages	–	[66]
TCONS_00019715	M1 macrophages	–	[75]

## Abbreviations

ACVR1B: Activin A receptor type 1B
AGO2: Argonaute 2
APL: Acute promyelocytic leukemia
ATRA: All trans retinoic acid
BMDM: Bone marrow-derived macrophages
CMPs: Common myeloid progenitors
DC: Dendritic cell
EGO: Eosinophil granule ontogeny
EPS: LincRNA-erythroid pro-survival
eRNAs: Enhancer RNAs
G-MDSCs: Granulocytic myeloid-derived suppressor cells
H3K27ac: Histone H3-lysine-27 acetylation
H3K4me3: Histone H3-lysine-4 trimethylation
HAT: Histone acetyltransferase
HOTAIR: HOX antisense intergenic RNA
HOTAIRM1: HOX antisense intergenic RNA myeloid 1
HOXA-AS2: HOXA cluster antisense RNA 2
HSCs: Hematopoietic stem cells
HSPCs: Hematopoietic stem/progenitor cells
HuR: Human antigen R
IFN-γ: Interferon gamma
IL: Interleukin
ITPR1: Inositol trisphosphate receptor type 1
LincRNAs: Long intergenic ncRNAs
LMPPs: Lymphoid-primed multi-potential progenitors
LncRNAs: Long non-coding RNAs
MEPs: Megakaryocyte-erythroid progenitors
miRNAs: MicroRNAs
Morrbid: Myeloid RNA regulator of Bim-induced death
MPPs: Multi-potential progenitors
ncRNA: Non-coding RNA
NEAT1: Nuclear paraspeckle assembly transcript 1
Pam3CSK4: Pam3-Cys-Ser-Lys4
PML: Promyelocytic leukemia
RA: Retinoic acid
RARα: Retinoic acid receptor α
TFs: Transcription factors
TRAIL: TNF-related apoptosis-inducing ligand
TSS: Transcription start site
